# Vulnerability profiles of workers and the relation with burnout symptoms: results from the Netherlands working conditions survey

**DOI:** 10.1007/s00420-024-02071-1

**Published:** 2024-05-27

**Authors:** Luuk Bouwens, Sander K.R. van Zon, Roy Peijen, Marloes Vooijs

**Affiliations:** 1 Amsterdam UMC location Vrije Universiteit Amsterdam, Public and Occupational Health, De Boelelaan 1117, Amsterdam, The Netherlands; 2 TNO Unit Healthy Living & Work, Netherlands Organisation for Applied Scientific Research, Sylviusweg 71, Leiden, 2333 BE The Netherlands; 3 Department of Health Sciences, Community and Occupational Medicine, University of Groningen, University Medical Center Groningen, Groningen, The Netherlands

**Keywords:** Burnout symptoms, Labor market, Work, Vulnerable, Populations, Work participation

## Abstract

**Introduction:**

Unfavorable working conditions may place workers in a vulnerable position in the labour market, but studies on the clustering of these factors and their relation to burnout symptoms are lacking. This study aims to identify subgroups of workers in potentially vulnerable positions in the labour market and examine whether burnout symptoms differ across the established subgroups.

**Methods:**

This study utilizes cross-sectional data from 2019 of the Netherlands Working Conditions Survey (n = 55,283). Working conditions included employment contracts, working hours, multiple jobs, tenure, physical strain, autonomy, and workload. Burnout symptoms were measured with five items on a 7-point Likert scale. Latent Class Analysis was used to identify vulnerability subgroups based on working conditions and educational level. Wilcoxon rank-sum tests were used to examine whether burnout symptoms differed between the identified subgroups.

**Results:**

Three out of nine subgroups (i.e., classes 4, 6, and 7) presented combinations of multiple unfavourable working conditions. The vulnerability of class 4, characterized by low educational level, physically demanding work, low autonomy, and a high workload, was underscored by a significantly higher burnout symptom score (M = 2.91;SD = 0.97) compared to all other subgroups. Subgroups 3 (M = 2.69;SD = 1.43) and 8 (M = 2.41;SD = 1.41), without striking unfavourable conditions, had the second and third highest scores on burnout symptoms.

**Conclusions:**

Determining vulnerability in the labour market is not straightforward as not all profiles that presented clusters of unfavourable working conditions scored high on burnout symptoms, and vice versa. Future research should investigate whether findings are similar to other mental health outcomes.

## Introduction

The value of work for individuals and society has been extensively demonstrated (Burgard and Lin 2013; Ryan and Deci 2000; van der Klink et al. 2016; Vooijs et al. 2018). Work participation provides structure and social interactions, fostering a sense of belonging and societal integration (Burgard and Lin 2013; van der Klink et al. 2016; Vooijs et al. 2018). Furthermore, engaging in *meaningful work* fulfils the fundamental psychological needs of autonomy, competence, and relatedness (Ryan and Deci 2000), all of which play a substantial role in an individual’s physical and mental health. An essential prerequisite for the positive effects of work participation is the quality of work and its environment itself, as it should meet certain standards of decency (Burgard and Lin 2013). Unfavourable working conditions may place workers in a vulnerable position in the labour market and lead to poor health outcomes like burnout (Datta Gupta and Kristensen 2008; Llena-Nozal 2009), being a vital mental health indicator for future disability pension (Ahola et al. 2009).

Vulnerability in the labour market pertains to indicators such as an unstable contract, poor compensation, long working hours (Julià et al. 2017; Koranyi et al. 2018; Vives et al. 2013), physical strain (Benach et al. 2014), high workload and low autonomy (Demerouti et al. 2001; Schaufeli and Bakker 2004; Niedhammer et al. 2021). However, in most cases, vulnerability is not determined by a single indicator but by a multitude or a specific combination of indicators (Vooijs et al. 2023). To the best of our knowledge, no studies have investigated the potential clustering of unfavourable working conditions and its relationship with burnout symptoms.

Previous studies have explored whether vulnerability typologies differ in general health (Fujishiro et al. 2021), mental health (Hasselhorn et al. 2020; Keller et al. 2017; van Aerden et al. 2016), emotional exhaustion (Keller et al. 2017), mental distress including stress, depression, and problems with emotions (Peckham et al. 2019), emotional problems (Vanroelen et al. 2010), and mental health disorders (Balogh et al. 2023). Moreover, several studies showed that unfavourable working conditions are related to burnout (Shahidi et al. 2021; Bouwhuis et al. 2019), but without examining clusters of unfavourable working conditions. In the pursuit of long and healthy working lives, it is essential to understand how unfavourable working conditions cluster and, subsequently, how they are related to health outcomes like burnout symptoms. This may offer stakeholders, like the government and employers, opportunities to adapt policies and support workers in preventing burnout.

Therefore, the first aim of this study is to identify subgroups of workers in potentially vulnerable positions in the labour market based on existing monitoring data from the Netherlands Working Conditions Survey. The second aim is to examine whether the degree of burnout symptoms differs across the established vulnerable subgroups.

## Methods

### Study design and sample

This study utilizes cross-sectional data from 2019 of the Netherlands Working Conditions Survey (NWCS) (*Nationale Enquête Arbeidsomstandigheden [NEA]* in Dutch) from the Netherlands Organisation for Applied Scientific Research (*Nederlandse Organisatie voor Toegepast Natuurwetenschappelijk Onderzoek [TNO]* in Dutch) (Hooftman et al. 2020). In the NWCS, a representative sample of the Dutch labour force is questioned annually about all aspects surrounding their job characteristics, work conditions, and sustainable employability. We used the annual dataset from 2019 (N = 58,316) for our secondary analyses to exclude COVID-19-related fluctuations in working conditions. The Ethics Committee of TNO approved the NWCS and assessed the NWCS as not being subject to the requirements of the Dutch Medical Research Involving Human Subjects Act (case 2018-066). Individuals aged 12 to 15 require parental/guardian consent to participate in the NWCS. This requirement is no longer applicable for individuals aged 16 and older.

### Measurements

#### Working conditions

Working conditions included employment contracts, weekly working hours, multiple jobs, tenure, physical strain, autonomy, and workload. *Employment contract* was self-reported and consisted of the categories [1] permanent contract (having prospects of); [2] fixed-term contract (temporary agreements); [3] on-call contract/no fixed hours (whether fixed-term or permanent). W*eekly working hours* was categorized as working [1] ≤ 40 h; [2] 41–48 h; [3] > 48 h. *Multiple jobs* was categorized as a dichotomous variable that indicates [1] one job; [2] multiple jobs. *Tenure* is represented by three categories indicating working [1] < 1 year; [2] 1–5 years; [3] 5 + years with the same employer.

*Physical strain* was assessed by five items in which respondents were asked to check the boxes linked to the following questions: (1) “Are you engaged in work that requires you to exert a lot of physical force, such as lifting, pushing, pulling, or carrying heavy objects? Or do you use tools or equipment in your job that require significant physical effort?”; (2) “Do you use tools, equipment, or vehicles in your work that produce vibrations or shaking?”; (3) “Do you perform work in an uncomfortable or awkward posture?”; (4) “Do you engage in work that involves repetitive movements?”; and (5) “Is there so much noise at your workplace that you have to speak loudly to be heard?” If one of the boxes has been checked, we speak of experiencing physical strain.

The perceived *autonomy* was assessed using the following five items on a 3-point scale (Karasek et al. 1998): (1) “Can you decide how to perform your work?”; (2) “Do you determine the order of your tasks yourself?”; (3) “Can you regulate your work pace on your own?”; (4) “In your work, do you need to come up with solutions for certain things yourself?”; (5) “Can you take leave when you want to?”. Participants could respond with: [1] No; [2] Yes, sometimes; and [3] Yes, regularly. To construct a dichotomous variable, we decided that a 3-item average of higher or equal to 2.5 represents perceiving [1] high autonomy, and any value below represents [2] low autonomy.

The perceived *workload*, also referred to as task demands, was assessed using the following three items: (1) “Do you have to work very quickly?”; (2) “Do you have to do a lot of work?”; (3) “Do you have to work extra hard?”. [3] Participants could respond with: [1] No; [2] Yes, sometimes; and [3] Yes, regularly. To construct a dichotomous variable, we decided that a 3-item average of higher or equal to 2.5 represents perceiving [1] high workload and any value below represents [2] low workload.

#### Educational level

*Educational level* consisted of the categories [1] low educated (primary, lower vocational and lower secondary education); [2] intermediate educated (intermediate vocational and intermediate secondary); [3] high educated (higher secondary, higher vocational and university).

#### Burnout symptoms

*Burnout symptoms* were measured by five items based on the validated Utrecht Burnout Scale (UBOS: Schaufeli and Van Dierendonck 2000), namely: (1) “I feel emotionally exhausted by my work”; (2) “At the end of a workday, I feel empty”; (3) “I feel tired when I wake up in the morning and face my work”; (4) “It takes a lot out of me to work with people all day”; (5) “I feel completely drained by my work”. Respondents were asked to answer these questions on a 7-point Likert scale where [1] never; [2] a few times a year; [3] monthly; [4] a few times a month; [5] every week; [6] a few times a week; [7] every day. The responses to the five items are averaged on a scale ranging from 1 to 7, where higher scores indicate a higher frequency of burnout symptoms.

#### Covariates

Gender, age, ethnicity, business size, occupation, and industry were included as covariates. Respondents’ ages at the time of the survey were grouped into six categories (15–24, 25–34, 35–44, 45–54, 55–64, and 65–75 years). Ethnicity was differentiated among Dutch natives, individuals of Western background, and those of non-Western background (1st and 2nd generation). Business size was segmented into small (1–49 employees), average (50–249 employees), and large (250 or more employees) categories. Additionally, occupational classifications adhered to the ISCO-08 Major categorization (International Labour Office 2008).

### Statistical analysis

First, descriptive statistics were presented for the total study population. Second, we performed Latent Class Analysis (LCA) in R using the poLCA package to classify individuals based on their working conditions. Individuals with any missing values on either of the characteristics of interest were dropped from the dataset (n = 3,033), keeping 55,283 individuals in our dataset. We implemented a loop of LCA analyses that determined the optimal number of classes (ranging from 2 to 10) based on a vector of job characteristics with the following options: maxiter = 5000, tol = 0.001, and nrep = 20. The optimal number of classes was determined by the lowest Bayesian information criterion (BIC) value as provided by the loop of LCAs testing for 2 to 10 categories. We chose the BIC over the Akaike Information Criterion (AIC) as the BIC tends to be a more reliable indicator of the number of classes in LCA than the AIC as the AIC does not correct estimates for the sample size being problematic with larger samples (Nylund et al., 2007). Finally, Wilcoxon rank-sum tests were applied to examine whether burnout symptoms differed between the identified classes.

## Results

### Description of the study sample

Most employees were 55–64 years old (23.9%), followed by 45–54-year-olds (22.2%) and 35–44-year-olds (18.2%) (Table 1). The sample comprises nearly equal proportions of men (48.7%) and women (51.3%). Most of the sample was highly educated (44.2%), followed by intermediate education (39.3%) and low education (16.5%). Dutch natives constitute the vast majority in our sample (85.1%), while only a small proportion consists of individuals with a Western (8.5%) and non-Western background (6.4%).

**Table 1:** Sample characteristics

	Total (n = 55,283)
	N (%)
Sex	
Male	26,908 (48.7%)
Female	28,375 (51.3%)
Age	
15–24 years	8547 (15.5%)
25–34 years	9677 (17.5%)
35–44 years	10,060 (18.2%)
45–54 years	12,282 (22.2%)
55–64 years	13,195 (23.9%)
65–75 years	1522 (2.8%)
Education	
Low	9124 (16.5%)
Intermediate	21,708 (39.3%)
High	24,451 (44.2%)
Ethnicity	
Dutch native	46,928 (84.9%)
Western (1st generation)	1643 (3.0%)
Western (2nd generation)	2450 (4.4%)
Non-western (1st generation)	2514 (4.5%)
Non-western (2nd generation)	1747 (3.2%)
Bussiness size	
Small business (1–49 employees)	21,413 (38.7%)
Average business (50–249 employees)	15,807 (28.6%)
Large business (250 + employees)	17,918 (32.4%)
Missing	145 (0.3%)
Occupation (ISCO-08)	
Armed forces occupations	156 (0.3%)
Managers	2847 (5.1%)
Professionals	18,674 (33.8%)
Technicians and associate professionals	9104 (16.5%)
Clerical support workers	5485 (9.9%)
Service and sales workers	9231 (16.7%)
Skilled agricultural, forestry, and fishery workers	458 (0.8%)
Craft and related trades workers	3191 (5.8%)
Plant and machine operators and assemblers	2208 (4.0%)
Elementary occupations	3696 (6.7%)
Missing	233 (0.4%)

### Vulnerability classes

**Fig. 1:**
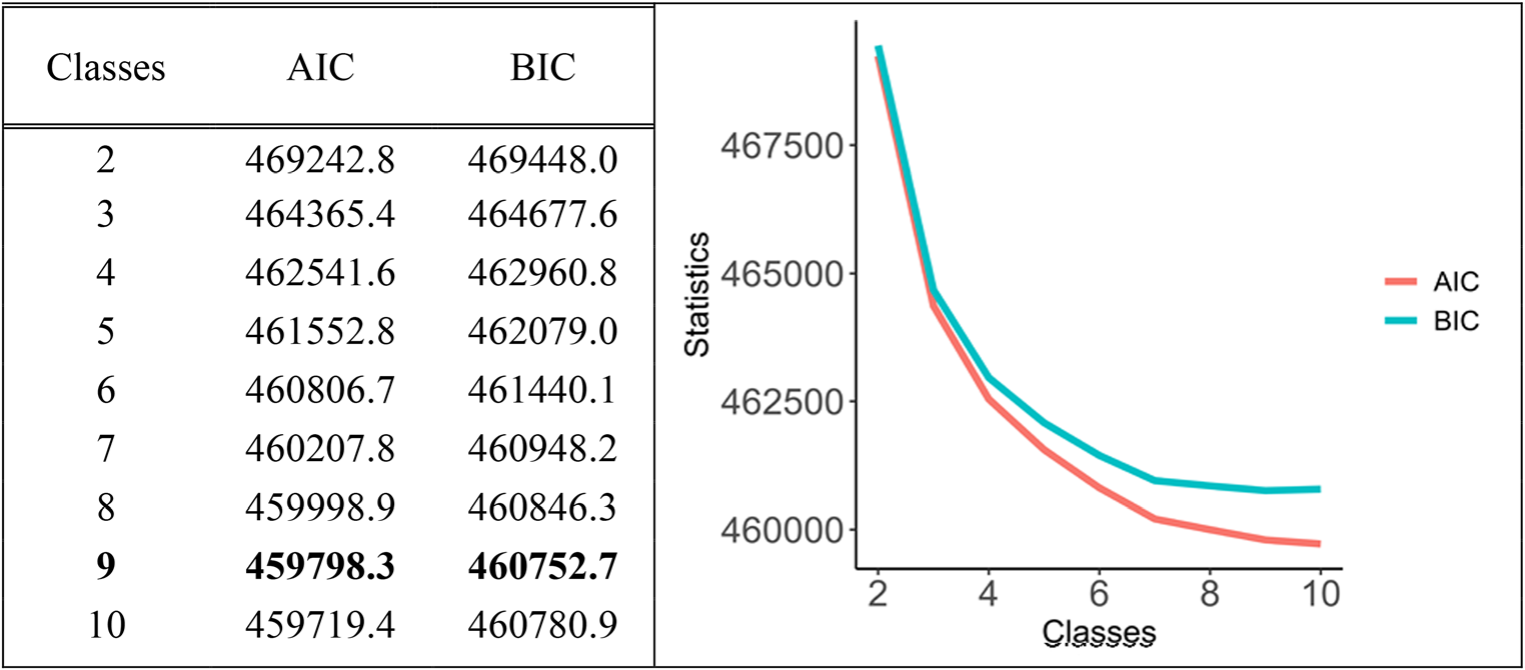
Number of classes and Akaike Information Criterion (AIC) and *Bayesian information criterion* (BIC) values. *The lowest BIC value determined the optimal number of classes found for nine classes (in bold). The optimal number of classes was determined by the lowest BIC value as provided by the loop of LCAs testing for 2 to 10 categories*

The lowest BIC value determined the optimal number of classes found for nine classes (Fig. 1). The classes ranged in size between 0.85% (n = 468) and 25.2% (n = 13,942) (Table 2). Table 2 shows the likelihood of an answer category for the employment terms and working conditions for each specific class to visualize the degree of potential vulnerability among the nine classes. Three classes (classes 4, 6 and 9) emerge where unfavourable employment terms and working conditions seem to cluster.

**Table 2:**
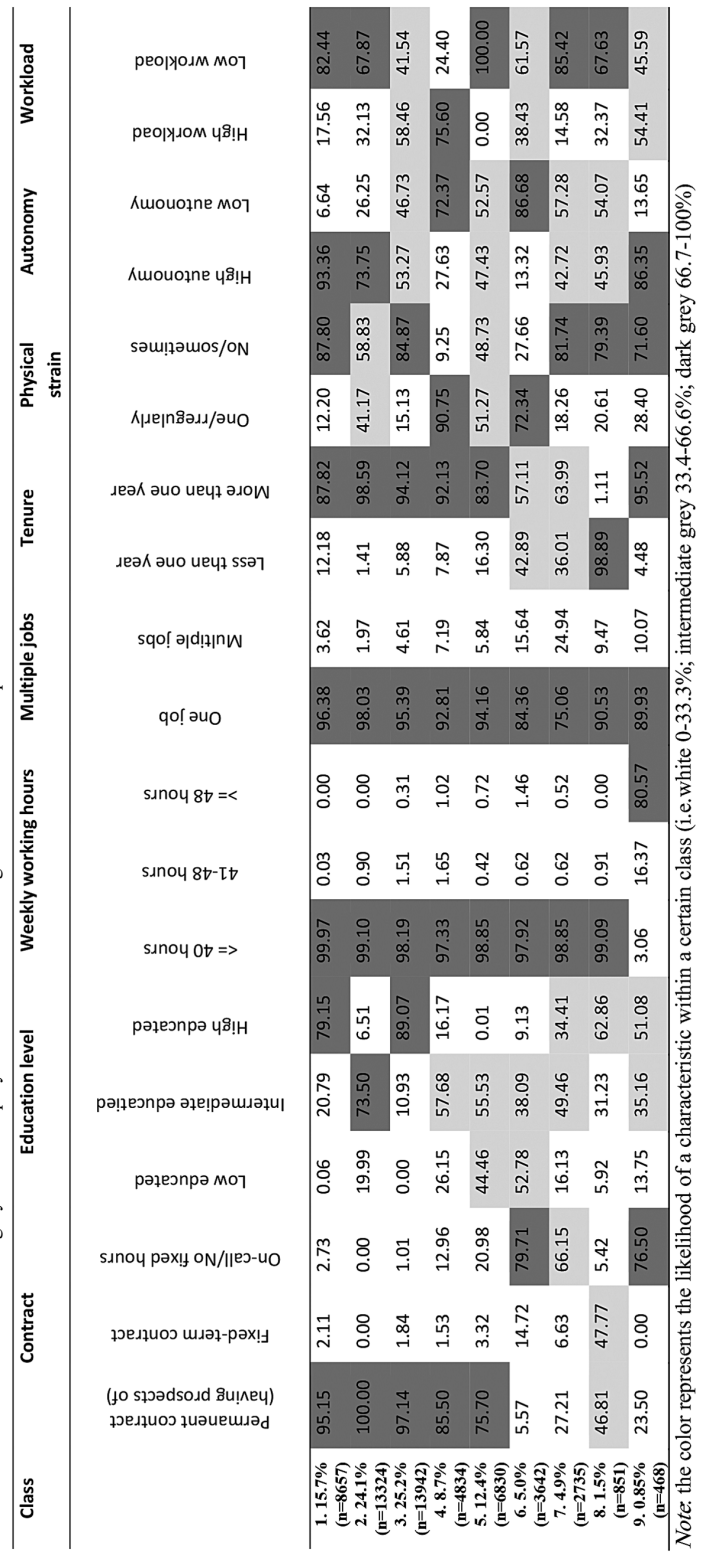
The chance of an answer category for the employment terms and working conditions for each specific class

Class 4 (N = 4,834) is characterized by primarily having employees with a low or intermediate educational level, physically demanding work, low autonomy and a high workload (Table 2). Table 3 further shows that a large proportion of employees in this group (43%) work in small companies (1–49 employees) and are employed in the sectors of healthcare and social services (24.8%), wholesale and retail trade (21.2%) and industry (12.7%). Class 6 (N = 3,642) is characterized by an on-call/no fixed hours contract, a tenure duration of less than one year, physically demanding work and low autonomy (Table 2). Employees in this class are primarily individuals aged 15–24 years (71%) and lower- (53%) or intermediately educated (38%) (Table 3). Service and sales workers- (38%) and elementary occupations (30%) are common professions within this class. Class 9 (N = 468) is characterized by on-call/no fixed hours contract workers working more than 48 h per week (81%), with an intermediate workload (54%) (Table 2). This class primarily comprises male employees (89%) from the occupational groups managers (29%) and professionals (e.g. civil engineering technicians, medical doctors, teachers etc.) (29%) (Table 3).

**Table 3:** Sample characteristics stratified by class

	Class 1	Class 2	Class 3	Class 4	Class 5	Class 6	Class 7	Class 8	Class 9
	N = 8657	N = 13,324	N = 13,942	N = 4834	N = 6830	N = 3642	N = 2735	N = 851	N = 468
	N (%)	N (%)	N (%)	N (%)	N (%)	N (%)	N (%)	N (%)	N (%)
Sex									
Male	4776 (55.2%)	7284 (54.7%)	5561 (39.9%)	2129 (44.0%)	3577 (52.4%)	1658 (45.5%)	1207 (44.1%)	302 (35.5%)	414 (88.5%)
Female	3881 (44.8%)	6040 (45.3%)	8381 (60.1%)	2705 (56.0%)	3253 (47.6%)	1984 (54.5%)	1528 (55.9%)	549 (64.5%)	54 (11.5%)
Age									
15–24 years	374 (4.3%)	709 (5.3%)	559 (4.0%)	955 (19.8%)	1692 (24.8%)	2581 (70.9%)	1398 (51.1%)	251 (29.5%)	28 (6.0%)
25–34 years	1983 (22.9%)	1718 (12.9%)	3446 (24.7%)	786 (16.3%)	753 (11.0%)	301 (8.3%)	362 (13.2%)	288 (33.8%)	40 (8.5%)
35–44 years	2130 (24.6%)	2362 (17.7%)	3364 (24.1%)	731 (15.1%)	867 (12.7%)	198 (5.4%)	174 (6.4%)	132 (15.5%)	102 (21.8%)
45–54 years	2064 (23.8%)	3778 (28.4%)	3156 (22.6%)	1041 (21.5%)	1451 (21.2%)	263 (7.2%)	284 (10.4%)	107 (12.6%)	138 (29.5%)
55–64 years	1889 (21.8%)	4323 (32.4%)	3197 (22.9%)	1229 (25.4%)	1800 (26.4%)	248 (6.8%)	302 (11.0%)	68 (8.0%)	139 (29.7%)
65–75 years	217 (2.5%)	434 (3.3%)	220 (1.6%)	92 (1.9%)	267 (3.9%)	51 (1.4%)	215 (7.9%)	5 (0.6%)	21 (4.5%)
Education									
Low	0 (0%)	1485 (11.1%)	0 (0%)	1531 (31.7%)	3808 (55.8%)	2073 (56.9%)	147 (5.4%)	17 (2.0%)	63 (13.5%)
Intermediate	417 (4.8%)	11,839 (88.9%)	69 (0.5%)	2984 (61.7%)	3022 (44.2%)	1323 (36.3%)	1581 (57.8%)	317 (37.3%)	156 (33.3%)
High	8240 (95.2%)	0 (0%)	13,873 (99.5%)	319 (6.6%)	0 (0%)	246 (6.8%)	1007 (36.8%)	517 (60.8%)	249 (53.2%)
Ethnicity									
Dutch native	7371 (85.1%)	11,688 (87.7%)	11,881 (85.2%)	4025 (83.3%)	5757 (84.3%)	2892 (79.4%)	2269 (83.0%)	653 (76.7%)	392 (83.8%)
Western (1st generation)	317 (3.7%)	269 (2.0%)	482 (3.5%)	132 (2.7%)	166 (2.4%)	131 (3.6%)	83 (3.0%)	41 (4.8%)	22 (4.7%)
Western (2nd generation)	416 (4.8%)	566 (4.2%)	645 (4.6%)	199 (4.1%)	276 (4.0%)	159 (4.4%)	123 (4.5%)	41 (4.8%)	25 (5.3%)
Non-western (1st generation)	299 (3.5%)	495 (3.7%)	550 (3.9%)	325 (6.7%)	410 (6.0%)	219 (6.0%)	121 (4.4%)	70 (8.2%)	25 (5.3%)
Non-western (2nd generation)	254 (2.9%)	306 (2.3%)	384 (2.8%)	152 (3.1%)	221 (3.2%)	241 (6.6%)	139 (5.1%)	46 (5.4%)	4 (0.9%)
Bussiness size									
Small business (1–49 employees)	2511 (29.0%)	5077 (38.1%)	4652 (33.4%)	2079 (43.0%)	3154 (46.2%)	1804 (49.5%)	1474 (53.9%)	365 (42.9%)	297 (63.5%)
Average business (50–249 employees)	2281 (26.3%)	3673 (27.6%)	4326 (31.0%)	1381 (28.6%)	1907 (27.9%)	1292 (35.5%)	626 (22.9%)	229 (26.9%)	92 (19.7%)
Large business (250 + employees)	3858 (44.6%)	4546 (34.1%)	4948 (35.5%)	1359 (28.1%)	1728 (25.3%)	527 (14.5%)	619 (22.6%)	255 (30.0%)	78 (16.7%)
Missing	7 (0.1%)	28 (0.2%)	16 (0.1%)	15 (0.3%)	41 (0.6%)	19 (0.5%)	16 (0.6%)	2 (0.2%)	1 (0.2%)
Occupation (ISCO-08)									
Armed forces occupations	23 (0.3%)	65 (0.5%)	17 (0.1%)	13 (0.3%)	25 (0.4%)	9 (0.2%)	3 (0.1%)	0 (0%)	1 (0.2%)
Managers	735 (8.5%)	615 (4.6%)	1129 (8.1%)	89 (1.8%)	56 (0.8%)	12 (0.3%)	63 (2.3%)	13 (1.5%)	135 (28.8%)
Professionals	5056 (58.4%)	2130 (16.0%)	9517 (68.3%)	410 (8.5%)	351 (5.1%)	124 (3.4%)	597 (21.8%)	355 (41.7%)	134 (28.6%)
Technicians and associate professionals	1596 (18.4%)	3238 (24.3%)	1740 (12.5%)	806 (16.7%)	878 (12.9%)	230 (6.3%)	386 (14.1%)	164 (19.3%)	66 (14.1%)
Clerical support workers	609 (7.0%)	2443 (18.3%)	644 (4.6%)	374 (7.7%)	718 (10.5%)	221 (6.1%)	348 (12.7%)	109 (12.8%)	19 (4.1%)
Service and sales workers	409 (4.7%)	2414 (18.1%)	641 (4.6%)	1485 (30.7%)	1903 (27.9%)	1367 (37.5%)	857 (31.3%)	134 (15.7%)	21 (4.5%)
Skilled agricultural, forestry, and fishery workers	22 (0.3%)	123 (0.9%)	22 (0.2%)	78 (1.6%)	140 (2.0%)	51 (1.4%)	13 (0.5%)	2 (0.2%)	7 (1.5%)
Craft and related trades workers	98 (1.1%)	1222 (9.2%)	73 (0.5%)	546 (11.3%)	907 (13.3%)	231 (6.3%)	68 (2.5%)	19 (2.2%)	27 (5.8%)
Plant and machine operators and assemblers	30 (0.3%)	495 (3.7%)	58 (0.4%)	400 (8.3%)	775 (11.3%)	259 (7.1%)	131 (4.8%)	11 (1.3%)	49 (10.5%)
Elementary occupations	58 (0.7%)	545 (4.1%)	71 (0.5%)	622 (12.9%)	1025 (15.0%)	1089 (29.9%)	243 (8.9%)	36 (4.2%)	7 (1.5%)
Missing	21 (0.2%)	34 (0.3%)	30 (0.2%)	11 (0.2%)	52 (0.8%)	49 (1.3%)	26 (1.0%)	8 (0.9%)	2 (0.4%)

### Burnout symptoms

**Fig. 2:**
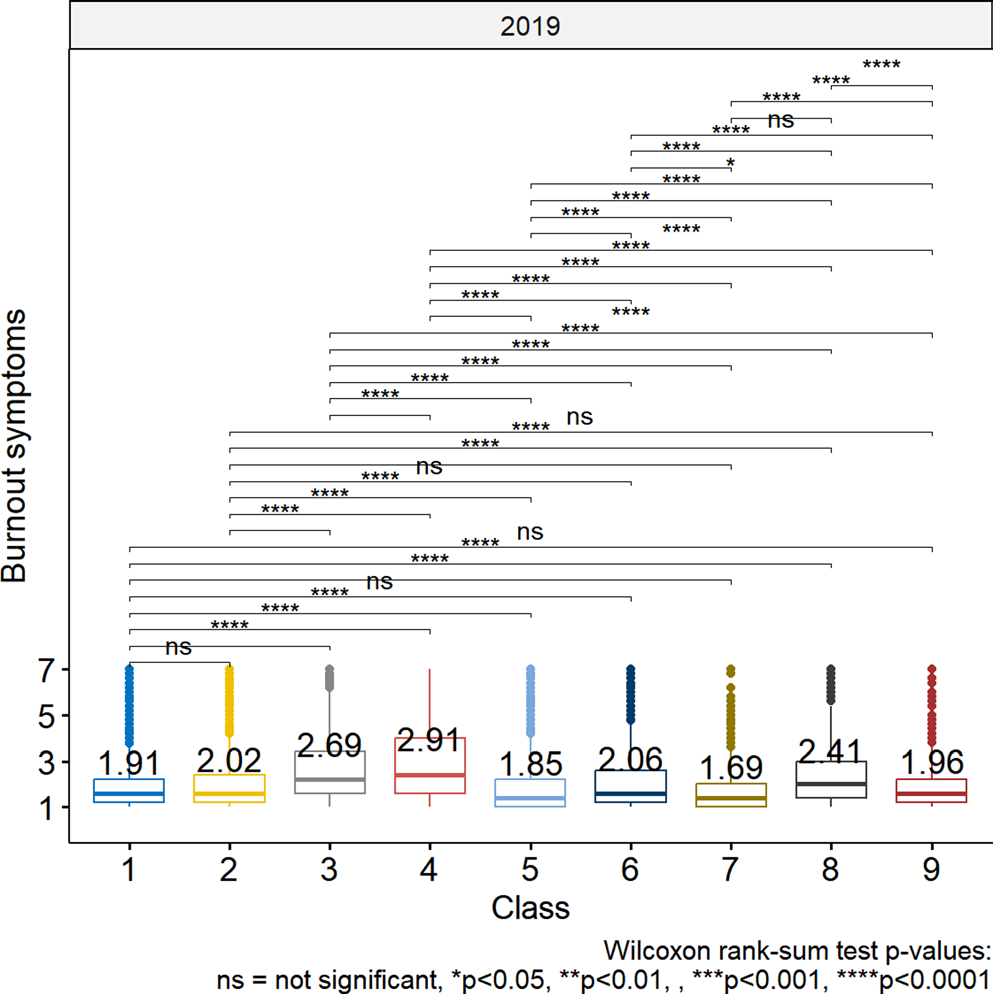
Burnout symptoms by vulnerability profile

Figure 2 shows the burnout symptoms by class. The asterisks displayed above the horizontal brackets show the statistically significant differences in burnout symptoms between classes based on Wilcoxon rank-sum tests. Employees from class 4, which was identified as one of the more vulnerable classes, scored significantly higher than all other classes on burnout symptoms (M = 2.91, SD = 0.97). Class 6 (M = 2.06, SD = 1.26) and class 9 (M = 1.96, SD = 1.18), which were previously characterized as potentially vulnerable classes, score relatively low on burnout symptoms. In contrast, class 3 (M = 2.69, SD = 1.43) and 8 (M = 2.41, SD = 1.41), which were not identified as potentially vulnerable classes, scored relatively high on burnout symptoms.

## Discussion

In this study, nine subgroups of workers with various degrees of potential vulnerability were identified based on their employment terms and working conditions. Three subgroups presented combinations of multiple unfavourable working conditions (i.e. class 4, 6, 7). The other subgroups were characterized by one or no unfavourable conditions. A significantly higher burnout symptom score underscored the vulnerability of class 4 compared to all other subgroups. However, burnout symptoms were not necessarily higher in classes that seemed most vulnerable based on their working conditions, as two subgroups without striking unfavourable conditions had the second and third highest scores on burnout symptoms.

Compared to previous studies, several similar typologies emerge. Typologies characterized by physically demanding work, in combination with low autonomy and/or high workload, are also found in previous studies (Eurofound 2016; Vanroelen et al. 2010). Similarly, distinct job typologies characterized by high job insecurity have been identified (Jonsson et al. 2021; Klug et al. 2019; Peckham et al. 2019), just as typologies characterized by practically no adverse job characteristics (Jonsson et al. 2021; Klug et al. 2019). In contrast to previous studies (Jonsson et al. 2021; Klug et al. 2019), flexible contracts and multi-job holding had limited discriminative power in determining distinct job typologies. This may be explained by the notion that compared to other countries, it is rather common in the Netherlands to have a flexible contract or hold multiple jobs (Eurostat 2023a, b).

While employees in class 4 and class 6 were similar regarding low educational level, high physical strain and low autonomy, they differed regarding having a high workload and, more importantly, regarding burnout symptoms. Given the answer categories of the UBOS, a mean value of 3 indicates monthly burnout symptoms. As we are looking at average group scores, this suggests that a substantial proportion of employees in class 4, with an average score of 2.91, may experience burnout symptoms monthly.

The relatively high score on burnout symptoms in class 4 compared to class 6 may be attributed to the combination of high job demands (physically demanding work and a high workload) and a lack of resources (low autonomy). Research has demonstrated that burnout is often a consequence of high job demands, i.e., aspects of work that require prolonged physical, emotional, or cognitive effort (Demerouti et al. 2001; Alarcon 2011). This effect is intensified by the absence of resources such as social support, autonomy, and skill variety (Bakker and Demerouti 2017; Lesener et al. 2019). An additional or alternative explanation is rooted in the demographic composition of class 6, predominantly comprising individuals aged 15 to 24 engaged in part-time on-call or no fixed-hour contracts working in service and sales and elementary occupations. Because these positions may pertain to part-time jobs held explicitly by students, which often entail limited working hours, this circumstance could explain why the combination of unfavourable conditions does not manifest in burnout symptoms.

Surprisingly, classes 3 and 8 exhibit relatively high scores on burnout symptoms, while these classes were not characterized by specific combinations of unfavourable working conditions. However, in class 3, most employees work in the education or healthcare and social services sector, where employees generally exhibit relatively high levels of burnout symptoms (Bridgeman et al. 2018; Garcia-Arroyo, Osca Segovia, & Peiró, 2019; van den Heuvel, Fernandez Beiro, & van Dam, 2022). The high score on burnout symptoms in class 8 also does not appear to be directly attributable to a specific combination of unfavourable working conditions, although almost all employees in this class have worked for the same employer for less than a year, which, in combination with a temporary contract, may contribute to job insecurity (Dekker and Schaufeli 1995).

Considering the findings of class 9, which also comprises a substantial number of highly educated individuals and simultaneously exhibits a relatively high score on burnout symptoms, it suggests a distinct manifestation of vulnerable conditions compared to the relatively lower-educated employees in classes 4 and 6. While in these classes, the predominant factors were physically demanding work and low autonomy, class 9 exhibits a substantial group of individuals who work 48 h or more per week and do this on-call or without fixed hours. Working long hours has previously been associated with poor mental health outcomes like burnout (Hu et al. 2016), which may be amplified by the fact that working hours were not fixed.

The current study’s strengths are the large sample size and representativeness of the Netherlands Working Condition Survey (NWCS) (Hooftman et al. 2020). Data from the NWCS enabled the exploration and identification of groups sharing a common set of unfavourable working conditions. By relating the identified classes with varying degrees of potential vulnerability to burnout symptoms, we also quantified the potential vulnerability first. A limitation of this study is the cross-sectional design, making it impossible to draw conclusions about causation. This study could not establish whether burnout symptoms are the result of specific combinations of working conditions or whether people with burnout symptoms are selected to work with unfavourable conditions. Second, we did not adjust the comparison of burnout symptoms between the identified classes for potential confounding variables. Therefore, future research should investigate whether our results hold when considering other factors like chronic health conditions. Third, there is some risk of information bias as all information is based on self-reports. Objective data on employment terms through registry data or medical diagnosis through linkage with healthcare providers may limit the risk of information bias in future studies.

Study results offer some leads for future research. Findings from this study indicate that determining vulnerability in the labour market is not straightforward, as not all profiles that presented clusters of unfavourable working conditions scored high on burnout symptoms, while profiles lacking such unfavourable clusters demonstrated elevated levels of burnout symptoms. Therefore, future research should investigate whether findings are similar for other mental health outcomes, which working conditions are indispensable for determining vulnerability, and which ones do not contribute substantially. Results from this study suggest, for example, that having a flexible contract and having multiple jobs does not contribute substantially to the classification of vulnerability profiles in the Netherlands. Additionally, future research could further explore the role of other potentially relevant working conditions by incorporating them into the classification, such as social relationships at work (e.g., support from colleagues, rewards, organizational justice) or emotional demands. Furthermore, future studies could examine whether potential vulnerability also translates to functioning at work (Abma et al. 2018). Moreover, burnout symptoms are distinct from the actual diagnosis of burnout. Currently, there is no consensus about a cut-off value for a burnout diagnosis. Therefore, more research is needed on the clinical relevance of these scores. Finally, future research could also qualitatively investigate what factors contribute to different groups having varying degrees of vulnerability and how this influences burnout symptoms.

## Conclusion

In conclusion, nine subgroups of workers were identified based on working conditions with a seemingly varying degree of vulnerability. The class characterized by having employees with a low or intermediate educational level, physically demanding work, low autonomy and a high workload scored highest on burnout symptoms. However, burnout symptoms were not necessarily higher in classes that seemed most vulnerable based on their working conditions, as two subgroups without striking unfavourable conditions had the second and third highest scores on burnout symptoms. This immediately highlights the main challenge for future research, which should further establish how vulnerable groups in the labour market can be better identified and how this relates to burnout symptoms and other mental health outcomes.

## Data Availability

Data may be obtained from a third party and are not publicly available. The conditions under which the NWCS is accessible are described by Statistics Netherlands (https://www.cbs.nl/en-gb/our-services/customised-services-microdata/microdata-conducting-your-own-research). Analysis scripts are available upon request.
